# Supplementary material for: The Contribution of Visual and Auditory Working Memory and Non-Verbal IQ to Motor Multisensory Processing in Elementary School Children
Source: Brain Sci. 2023 Feb 5;13(2):270. doi: 10.3390/brainsci13020270 (PMC9953899; doi:10.3390/brainsci13020270)
Supplement: Supplementary file 1 [file brainsci-13-00270-s001.zip › brainsci-2128585-supplementary.pdf]

Supplementary Material

The Contribution of Visual and Auditory Working Memory and Non-Verbal IQ to Motor Multisensory Processing in Early Elementary School Children

Areej A. Alhamdan [*], Melanie J. Murphy, Hayley E. Pickering & Sheila G. Crewther*

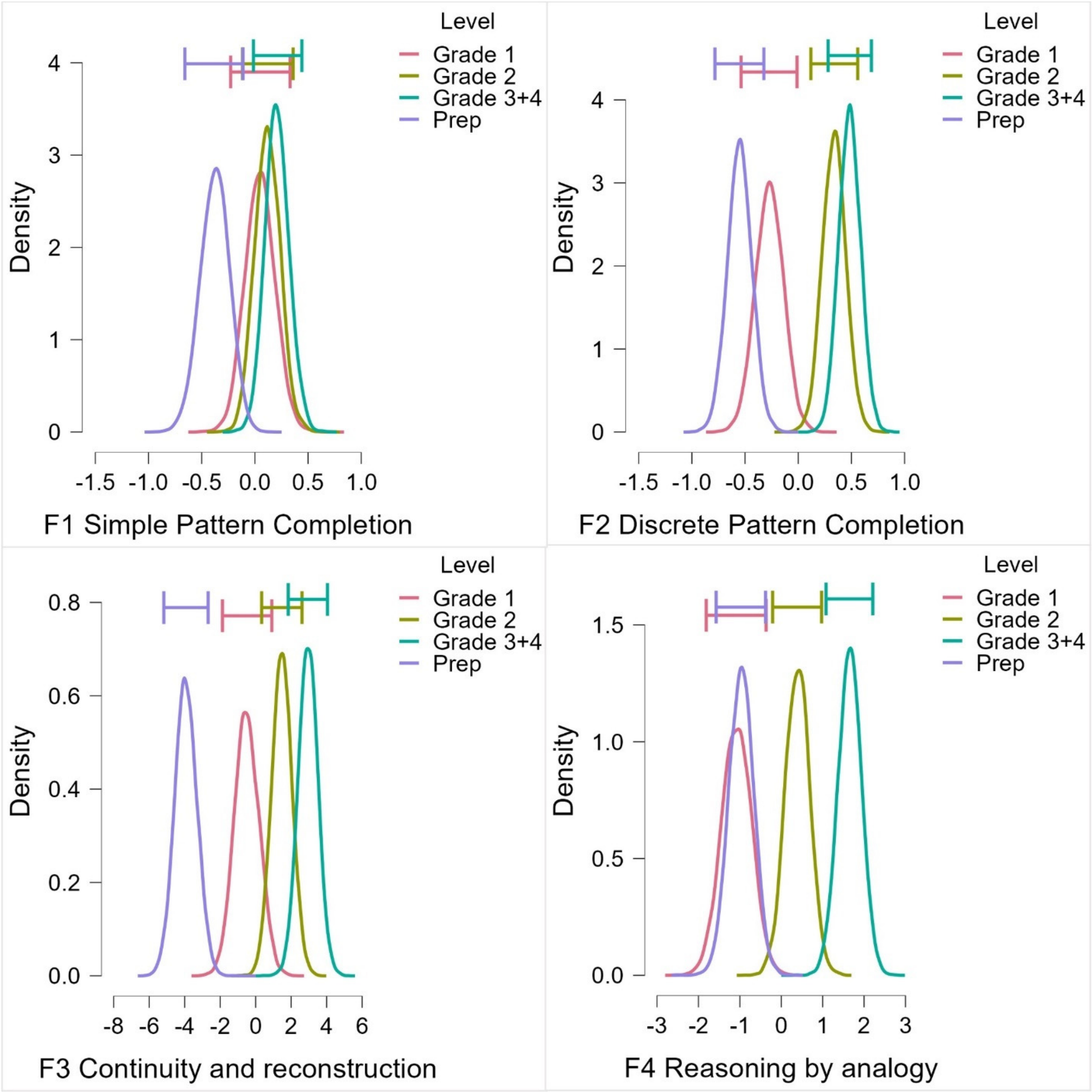

Supplementary Figure S1. The model-averaged posterior distribution (horizontal bars show the 95% credible intervals around the median) in Nonverbal IQ across four factors: Factor 1: Simple continuous pattern completion; Factor 2: Discrete pattern completion; Factor 3: Continuity and reconstruction of Simple and complex structures and Factor 4: Reasoning by analogy.

Supplementary Table S1. Bayesian Post Hoc comparisons in Nonverbal IQ across four factors: Factor 1 (SPC): Simple continuous pattern completion; Factor 2 (DPC): Discrete pattern completion; Factor 3 (ContRecon): Continuity and reconstruction of Simple and complex structures and Factor 4 (Reasoning): Reasoning by analogy.

		Prior Odds	Posterior Odds	$BF_{10,U}$	error %
**A. *Factor 1 (SPC)***					
**Grade 1**	Grade 2	0.414	0.169	0.408	0.003
	Grade 3+4	0.414	1.049	2.533	0.006
	Prep	0.414	0.29	0.701	0.003
**Grade 2**	Grade 3+4	0.414	0.367	0.886	0.007
	Prep	0.414	0.726	1.753	0.007
**Grade 3+4**	Prep	0.414	3.101	7.486	1.11e-06
**B. *Factor 2 (DPC)***					
**Grade 1**	Grade 2	0.414	3.317	8.008	8.93e-06
	Grade 3+4	0.414	292.493	706.141	5.47e-08
	Prep	0.414	0.2	0.482	0.003
**Grade 2**	Grade 3+4	0.414	0.69	1.666	0.008
	Prep	0.414	202.17	488.081	5.42e-08
**Grade 3+4**	Prep	0.414	154718.795	373524.213	6.82e-11
**C. *Factor 3 (ContRecon)***					
**Grade 1**	Grade 2	0.414	0.62	1.497	0.005
	Grade 3+4	0.414	18.784	45.349	4.55e-07
	Prep	0.414	1.568	3.785	8.30e-06
**Grade 2**	Grade 3+4	0.414	0.774	1.868	0.008
	Prep	0.414	2680.528	6471.368	1.40e-09
**Grade 3+4**	Prep	0.414	4.21e+06	1.02e+07	3.09e-12
**D. *Factor 4 (Reasoning)***					
**Grade 1**	Grade 2	0.414	3.105	7.496	9.93e-06
**Grade 2**	Grade 3+4	0.414	2063.474	4981.667	1.18e-09
	Prep	0.414	0.161	0.389	0.002
	Grade 3+4	0.414	1.695	4.093	1.05e-06
	Prep	0.414	4.207	10.158	2.37e-06
**Grade 3+4**	Prep	0.414	22233.81	53677.163	1.42e-10

Supplementary Table S2. Bayesian Pearson Correlations (Prep)

**Variable**		**Age**	**RCPM**	**AS**	**VS**	**AVS**	**SLURP**	**VDSF**	**VDSB**	**ADSF**	**ADSB**
1. Age	Pearson's r	—									
	BF_{10}	—									
2. RCPM	Pearson's r	0.618	—								
	BF_{10}	7.497	—								
3. AS	Pearson's r	-0.070	0.219	—							
	BF_{10}	0.310	0.417	—							
4. VS	Pearson's r	-0.008	0.443	0.375	—						
	BF_{10}	0.300	1.296	0.829	—						
5. AVS	Pearson's r	-0.225	0.267	0.771 ***	0.547	—					
	BF_{10}	0.426	0.493	124.152	3.248	—					
6. SLURP	Pearson's r	-0.437	-0.139	0.326	0.460	0.516	—				
	BF_{10}	1.158	0.348	0.621	1.355	2.124	—				
7. VDSF	Pearson's r	0.310	0.547	-0.236	-0.136	-0.075	-0.511	—			
	BF_{10}	0.554	1.864	0.449	0.373	0.350	1.297	—			
8. VDSB	Pearson's r	-0.331	-0.028	0.458	0.325	0.446	-0.033	-0.029	—		
	BF_{10}	0.594	0.342	1.050	0.581	0.987	0.356	0.342	—		
9. ADSF	Pearson's r	-0.198	0.357	0.232	-0.161	0.509	0.046	0.622	0.068	—	
	BF_{10}	0.413	0.655	0.445	0.387	1.428	0.358	2.869	0.362	—	
10. ADSB	Pearson's r	-0.066	0.390	-0.042	-0.071	0.311	-0.304	0.514	0.225	0.647	—
	BF_{10}	0.361	0.721	0.357	0.362	0.550	0.536	1.328	0.444	3.614	—

* $BF_{10} > 10$, ** $BF_{10} > 30$, *** $BF_{10} > 100$

Note. Age = age in numbers; RCPM = nonverbal IQ of Raven; AS = MRT of auditory stimuli; VS = MRT of visual stimuli; AVS = MRT of audiovisual stimuli; SLURP= visual motor skills; VDSF = visual digit span forward; VDSB = visual digit span backward; ADSF = auditory digit span forward; ADSB = auditory digit span backward.

Supplementary Table S3. Bayesian Pearson Correlations (Grade1)

**Variable**		**Age**	**RCPM**	**AS**	**VS**	**AVS**	**SLURP**	**VDSF**	**VDSB**	**ADSF**	**ADSB**
1. Age	Pearson's r	—									
	BF_{10}	—									
2. RCPM	Pearson's r	0.561	—								
	BF_{10}	1.558	—								
3. AS	Pearson's r	-0.091	-0.045	—							
	BF_{10}	0.381	0.372	—							
4. VS	Pearson's r	-0.147	0.002	0.673	—						
	BF_{10}	0.402	0.369	3.645	—						
5. AVS	Pearson's r	-0.198	0.071	0.832 **	0.789 *	—					
	BF_{10}	0.431	0.377	30.644	14.567	—					
6. SLURP	Pearson's r	0.419	0.195	-0.403	-0.560	-0.242	—				
	BF_{10}	0.739	0.440	0.702	1.360	0.473	—				
7. VDSF	Pearson's r	-0.016	-0.035	0.070	0.043	-9.823e-4	0.247	—			
	BF_{10}	0.370	0.371	0.377	0.372	0.369	0.478	—			
8. VDSB	Pearson's r	-0.300	0.198	0.033	0.020	-0.041	-0.233	0.375	—		
	BF_{10}	0.530	0.431	0.371	0.370	0.372	0.467	0.660	—		
9. ADSF	Pearson's r	-0.238	-0.198	-0.305	-0.097	-0.175	0.120	0.018	0.179	—	
	BF_{10}	0.462	0.431	0.537	0.383	0.417	0.406	0.370	0.418	—	
10. ADSB	Pearson's r	-0.021	-0.281	-0.221	0.068	-0.339	-0.295	-0.090	-0.239	-0.418	—
	BF_{10}	0.370	0.507	0.448	0.376	0.589	0.525	0.381	0.463	0.772	—

* $BF_{10} > 10$, ** $BF_{10} > 30$, *** $BF_{10} > 100$

Note. Age = age in numbers; RCPM = nonverbal IQ of Raven; AS = MRT of auditory stimuli; VS = MRT of visual stimuli; AVS = MRT of audiovisual stimuli; SLURP= visual motor skills; VDSF = visual digit span forward; VDSB = visual digit span backward; ADSF = auditory digit span forward; ADSB = auditory digit span backward.

Supplementary Table S4. Bayesian Pearson Correlations (Grade2)

**Variable**		**Age**	**RCPM**	**AS**	**VS**	**AVS**	**SLURP**	**VDSF**	**VDSB**	**ADSF**	**ADSB**
1. Age	Pearson's r	—									
	BF_{10}	—									
2. RCPM	Pearson's r	-0.111	—								
	BF_{10}	0.301	—								
3. AS	Pearson's r	0.289	0.024	—							
	BF_{10}	0.576	0.272	—							
4. VS	Pearson's r	0.469	-0.305	0.679 **	—						
	BF_{10}	2.342	0.630	57.542	—						
5. AVS	Pearson's r	0.256	-0.387	0.610 *	0.856 ***	—					
	BF_{10}	0.486	1.102	15.360	23255.394	—					
6. SLURP	Pearson's r	-0.221	0.008	-0.074	-0.050	-0.157	—				
	BF_{10}	0.425	0.318	0.329	0.323	0.367	—				
7. VDSF	Pearson's r	-0.184	-0.215	-0.138	-0.186	-0.103	-0.348	—			
	BF_{10}	0.364	0.408	0.319	0.368	0.297	0.670	—			
8. VDSB	Pearson's r	-0.197	0.213	0.389	0.322	0.264	-0.319	0.346	—		
	BF_{10}	0.381	0.404	1.127	0.699	0.507	0.590	0.819	—		
9. ADSF	Pearson's r	0.118	0.190	0.389	-0.044	-0.031	-0.393	0.258	0.311	—	
	BF_{10}	0.306	0.372	1.120	0.275	0.273	0.836	0.492	0.655	—	
10. ADSB	Pearson's r	-0.077	0.325	0.185	-0.025	-0.128	-0.527	0.207	0.314	0.093	—
	BF_{10}	0.285	0.711	0.366	0.272	0.312	2.066	0.395	0.667	0.291	—

* $BF_{10} > 10$, ** $BF_{10} > 30$, *** $BF_{10} > 100$

Note. Age = age in numbers; RCPM = nonverbal IQ of Raven; AS = MRT of auditory stimuli; VS = MRT of visual stimuli; AVS = MRT of audiovisual stimuli; SLURP= visual motor skills; VDSF = visual digit span forward; VDSB = visual digit span backward; ADSF = auditory digit span forward; ADSB = auditory digit span backward.

Supplementary Table S5. Bayesian Pearson Correlations (Grade 3+4)

**Variable**		**Age**	**RCPM**	**AS**	**VS**	**AVS**	**SLURP**	**VDSF**	**VDSB**	**ADSF**	**ADSB**
1. Age	Pearson's r	—									
	BF_{10}	—									
2. RCPM	Pearson's r	0.261	—								
	BF_{10}	0.484	—								
3. AS	Pearson's r	-0.448	-0.510	—							
	BF_{10}	1.467	2.547	—							
4. VS	Pearson's r	-0.494	-0.653 *	0.833 ***	—						
	BF_{10}	2.189	15.887	1362.412	—						
5. AVS	Pearson's r	-0.555	-0.452	0.689 *	0.814 ***	—					
	BF_{10}	4.120	1.519	29.833	699.800	—					
6. SLURP	Pearson's r	-0.414	0.026	0.466	0.211	0.407	—				
	BF_{10}	1.130	0.293	1.695	0.405	1.078	—				
7. VDSF	Pearson's r	0.257	0.262	-0.548	-0.255	-0.102	-0.367	—			
	BF_{10}	0.477	0.486	3.820	0.474	0.314	0.829	—			
8. VDSB	Pearson's r	0.410	0.573	-0.600	-0.468	-0.310	-0.365	0.672 *	—		
	BF_{10}	1.102	5.134	7.176	1.733	0.604	0.819	21.683	—		
9. ADSF	Pearson's r	0.575	0.668 *	-0.591	-0.590	-0.541	-0.452	0.446	0.790 ***	—	
	BF_{10}	5.220	20.282	6.375	6.368	3.513	1.508	1.437	326.280	—	
10. ADSB	Pearson's r	0.272	0.658 *	-0.332	-0.280	-0.134	-0.140	0.539	0.650 *	0.544	—
	BF_{10}	0.508	17.100	0.675	0.524	0.332	0.336	3.461	15.077	3.645	—

* $BF_{10} > 10$, ** $BF_{10} > 30$, *** $BF_{10} > 100$

Note. Age = age in numbers; RCPM = nonverbal IQ of Raven; AS = MRT of auditory stimuli; VS = MRT of visual stimuli; AVS = MRT of audiovisual stimuli; SLURP= visual motor skills; VDSF = visual digit span forward; VDSB = visual digit span backward; ADSF = auditory digit span forward; ADSB = auditory digit span backward.
